# Multiplex Degenerate Primer Design for Targeted Whole Genome Amplification of Many Viral Genomes

**DOI:** 10.1155/2014/101894

**Published:** 2014-08-03

**Authors:** Shea N. Gardner, Crystal J. Jaing, Maher M. Elsheikh, José Peña, David A. Hysom, Monica K. Borucki

**Affiliations:** ^1^Computations, Lawrence Livermore National Laboratory (LLNL), Livermore, CA 94550, USA; ^2^Physical and Life Sciences/Global Security, Lawrence Livermore National Laboratory (LLNL), Livermore, CA 94550, USA

## Abstract

*Background*. Targeted enrichment improves coverage of highly mutable viruses at low concentration in complex samples. Degenerate primers that anneal to conserved regions can facilitate amplification of divergent, low concentration variants, even when the strain present is unknown. *Results*. A tool for designing multiplex sets of degenerate sequencing primers to tile overlapping amplicons across multiple whole genomes is described. The new script, run_tiled_primers, is part of the PriMux software. Primers were designed for each segment of South American hemorrhagic fever viruses, tick-borne encephalitis, Henipaviruses, Arenaviruses, Filoviruses, Crimean-Congo hemorrhagic fever virus, Rift Valley fever virus, and Japanese encephalitis virus. Each group is highly diverse with as little as 5% genome consensus. Primer sets were computationally checked for nontarget cross reactions against the NCBI nucleotide sequence database. Primers for murine hepatitis virus were demonstrated in the lab to specifically amplify selected genes from a laboratory cultured strain that had undergone extensive passage in vitro and in vivo. *Conclusions*. This software should help researchers design multiplex sets of primers for targeted whole genome enrichment prior to sequencing to obtain better coverage of low titer, divergent viruses. Applications include viral discovery from a complex background and improved sensitivity and coverage of rapidly evolving strains or variants in a gene family.

## 1. Background

Sequencing whole genomes of potentially heterogeneous or divergent viruses can be challenging from a small or complex sample with low viral concentrations. Deep sequencing to detect rare viral variants or metagenomic sequencing to genotype viruses from a complex background requires targeted viral amplification. Techniques such as consensus PCR, Ion Ampliseq (Life Technologies) [1], TruSeq Amplicon (Illumina), and Haloplex (Agilent) [2] apply highly multiplexed PCR for target enrichment. Targeted enrichment should preferentially amplify the target virus over host or environmental DNA/RNA, in contrast to random amplification commonly used prior to whole genome sequencing. Primers designed to tile amplicons across a set of related viral genomes prior to sequencing can enrich whole viral genomes or large regions. However, high levels of intraspecific sequence variation combined with low virus concentrations mean that standard PCR primer design from a reference may fail due to mutations in the sample virus that prevent primer binding. To address this problem, we added a capability to the PriMux software distribution (http://sourceforge.net/projects/primux/) called run_tiled_primers that applies the PriMux software [3] to automate PCR primer design to achieve a near-minimal set of conserved, degenerate, multiplex-compatible primers designed to tile overlapping regions across multiple related whole genomes or regions.

JCVI has an automated degenerate PCR primer design system called JCVI Primer Designer, which is similar to run_tiled_primers in that it designs degenerate primers to tile across viral genomes [4]. The major difference is that it begins with a consensus sequence containing degenerate bases and selects primers with fewer than 3 or 4 degenerate bases, so that in the end a majority of strains are amplified, but it does not require primers to amplify all strains. In their examples, most of the primer pairs could amplify >75% of isolates. Each primer pair for a given region is intended to be run as a specific pair, not as a multiplex with multiple pairs. Consensus sequences with too little conservation, that is, <90% consensus, are divided manually in a preprocessing step into subgroups which can be run separately through the pipeline. The method here differs in that it takes the full multiple sequence alignment as input rather than a consensus, and it seeks to automatically design a minimal, degenerate set of multiplex compatible primers to amplify all the strains for a given region in a single reaction. The major operational difference of run_tiled_primers compared to the JCVI pipeline is that run_tiled_primers does not require manual subdivision of the target sequences into high consensus groups to be run separately by the user, and run_tiled_primers attempts to cover 100% of the target sequences in a single pass using a greedy minimal set algorithm.

Some regions of high conservation may have only one primer pair predicted to amplify all strain variants, while other regions may require many primers to cover all known variants. If multiple strains are present at once or if multiple forward and/or reverse primers in the multiplex amplify the strain present, the reaction will generate multiple overlapping amplicons spanning the same region, which could be problematic if exactly one amplicon sequence is needed, for example, for Sanger sequencing. In this case, the JCVI Primer Designer would be preferable since it designs primer pairs each to be run in singleplex reactions rather than as a multiplex, with the risk that outlier strains may not be amplified. However, when multiple overlapping reads with different endpoints or from different strains are acceptable, as in high throughput sequencing, run_tiled_primers should be suitable and could serve as a good alternative to random amplification when more specific enrichment is needed, and amplification of outliers is desired.

For the viral groups we used here, the target sets included up to hundreds of sequences, and in many cases consensus was extremely low, as little as 5% of the bases in the multiple sequence alignment (Table 1). The JCVI Primer Designer pipeline with a manual approach of subdividing the sequences into groups with 90% consensus and running each group separately could be a labor-intensive endeavor and would certainly result in a large number of singleplex reactions to cover each genome.

Possible applications include target enrichment for viral discovery of new members in a viral family from a complex host background, improving high throughput sequencing sensitivity and coverage of a rapidly evolving virus, or enriched coverage of variants in a gene family. We demonstrate the scalability of this software for designing whole genome amplification primers for a number of highly pathogenic viral groups which display very high levels of sequence variation, and for which we anticipate that targeted enrichment would be needed to obtain adequate sensitivity and genome coverage when sequencing from a clinical or environmental sample.

## 2. Implementation

### 2.1. Process

**Figure 1:**
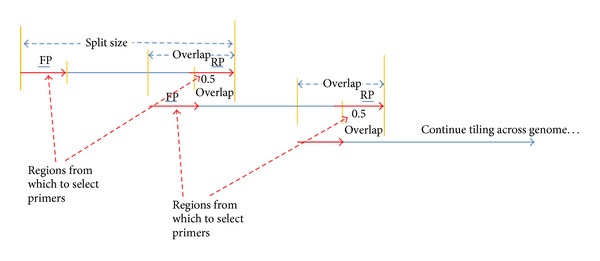
Diagram showing how the multiple sequence alignment is split into overlapping sections, and conserved; degenerate sets of primers are designed near the ends of the overlapping pieces so that overlapping amplicons should be produced which tile across the viral genome. FP = forward primer; RP = reverse primer.

The run_tiled_primers process can be summarized as follows: split a multiple sequence alignment into overlapping regions, and for each region design a degenerate multiplex set of primers that in combination amplify that region in all strains with as few primers as possible. Run_tiled_primers takes as input a multiple sequence alignment (MSA). Run_tiled_primers splits the alignment into regions of size “*s*” bases that overlap by “*x*” bases (Figure 1).

When splitting the alignment into regions of size *s*, if the last “remainder” piece of an alignment is less than half of *s*, then *s* is increased by the amount that evenly divides the alignment without any remainder to *s*′, and the split regions are recalculated with *s*′. If a user desires to tile across only selected regions instead of tiling across the entire sequence, then an optional regions file may be specified which contains the regions (e.g., genes) and their start and end positions in the alignment.

For each region, the PriMux software [3] is used to search for conserved, degenerate, and multiplex compatible primer sets to amplify that region in all target sequences with as few primers as possible. The PriMux “max” algorithm is used. Primers should be multiplex compatible since the primers for a given region are predicted not to form primer dimers and all to have *T*_*m*_'s in a range specified by the user. As run_tiled_primers is a wrapper script around the PriMux workhorse, all the primer design characteristics are specified in a PriMux options file. The minimum and maximum amplicon lengths are determined by the (*s*, *x*) parameters to run_tiled_primers (Table 2), so these parameters may be omitted in the input options file or if they are present, their values will be replaced with values appropriate for the specified values of (*s*, *x*). Run_tiled_primers requires that primers must anneal within 0.5*x* of either end of the region. If the value of *x* is 36 bp or less, it is too short for two nonoverlapping primers, typically at least 18 bp long. In this case, the code does not require that adjacent regions overlap and amplicons are allowed from anywhere in each region. Small overlaps (e.g., 40–80) do not leave much room to find good priming regions that pass the filters on *T*_*m*_, entropy, free energy, and homopolymers as specified in the options file, and consequently it may not be possible to find primers for all targets. When this happens, increasing the overlap and relaxing the primer specifications may be necessary.

Requiring that primers fall within 0.5*x* bases of the ends of each region facilitates the creation of amplicons which should overlap across a genome, allowing full genome assembly from the amplified products. There may not be amplicons covering the extreme 5′ and 3′ ends of a target sequence, since the first and last primers may be located some distance (maximum of *x*/2) from the ends. Rapid Amplification of cDNA Ends (RACE) PCR would be necessary to amplify the genome ends not covered by an overlapping region, priming with the reverse complement of the run_tiled_primers primers closest to the end so as to prime toward the edge of the genome.

Because this split size is based on the alignment and since dashes in the alignment are not counted in amplicon length, actual amplicons may be substantially shorter than the split size *s*. This is likely to happen for poorly aligning regions or regions in which there are insertions or deletions in a subset of the sequences. To compensate for this, one should select *s* that is larger than the actual amplicon lengths desired, particularly if the length of the MSA is much larger than the average genome length.

Run_tiled_primers labels each overlapping region as #part, where # indicates the order of the regions, for example, 0part, 1part, and 2part are the three regions shown in Figure 1. For each region, sets of conserved, degenerate primers are designed to ensure amplification of all the targets, if possible, given the primer specifications.

The primers can be run in separate singleplex reactions for each split region, or, alternatively, primers for all regions can be combined in a large multiplex after the large set is checked for primer dimers that could occur between primers from different regions. Combining primers for all regions in multiplex should facilitate whole genome amplification in a single reaction. It may yield longer amplicons from the reaction of forward and reverse primers from different parts (FP from 0part reacting with RP from 1part gives product ~2 times the split size), depending on the polymerase processivity and the duration of the extension step, and should facilitate assembly across amplified regions. This helps alleviate cases where a primer cannot be found for one part in an outlier genome due to *T*_*m*_, homopolymers, primer dimer Δ*G*, and so forth, since primers from different parts may amplify across the region. However, since primers of overlapping regions can also produce amplicons shorter (less than *x* bp) than the desired amplicon of length between *s* − *x* and *s* bp (e.g., RP of 0part with the FP from 1part), a step to remove short amplicons before sequencing may be desired. In our experimental test with MHV, the primers from parts 0, 2, and 4 were combined in one reaction and the primers from parts 1 and 3 were combined in another, so that short products would not be produced.

We used the script simulate_PCR.pl (https://sourceforge.net/projects/simulatepcr/ [7]) to predict all PCR amplicons from the multiplex degenerate primers compared to the target sequences and to the NCBI nt database. This script is run automatically from the run_tiled_primers code after it predicts primers. It is set to predict amplicons up to twice the maximum amplicon length specified by the user.

### 2.2. Computational Examples

Computationally predicted tiled primer sets were generated for the viruses and primer specifications provided in Table 1. MSAs were created with MUSCLE [5]. Two settings of split size *s* and overlap size *x* were used: long amplicons with *s* = 10,000, *x* = 500; or short amplicons of *s* = 3000, *x* = 500. The choice of which set to use could depend upon the product lengths the polymerase can amplify and the duration of the extension step of PCR. These fairly long amplicons are provided as theoretical examples. Users may run run_tiled_primers with shorter amplicons (e.g., *s* = 400 bp) to divide the MSA into many more parts. One amplicon per target sequence per region was desired (PriMux option file with - primer_selection_iterations = 1). Table 1 shows the average genome or segment length, the number of genomes available for each target, the % consensus among those sequences, and the total number of primers to amplify all overlapping regions of all genomes. All products from the nt database under 7800 bp (shorter amplicon) or 26 kb (longer amplicon) were predicted with simulate_PCR to identify potential amplification of nontarget organisms (Tables 3 and 4).

### 2.3. Murine Hepatitis Virus Example

Run_tiled_primers was used to design primers for selected regions of the coronavirus murine hepatitis virus (strain MHV-1) genome following passage in the lab, for a separate project in which deep sequencing of selected regions following lab passage was performed. In other work attempting to amplify passaged RNA viruses, finding robust primers based on the original genome was difficult due to mutations which modified primer binding sites [8]. It was hoped that run_tiled_primers would help avoid selecting primers in mutational hotspots by taking into account strain variation across multiple available genomes for the species, since run_tiled_primers seeks maximally conserved primers in the available sequences.

Input to run_tiled_primers was an alignment of 22 MHV genomes (genome identities provided as supplementary information) created using MUSCLE [5]. Regions tiled were the Nsp1, Nsp3, Nsp14, and several genes at the 3′ end of the genome (regions file provided in supplementary information), using the primer parameters in Table 2. Primer sets were predicted to produce overlapping amplicons for these regions from all MHV genomes, and a subset of primers predicted to amplify the MHV-1 or MHV strain JHM genome was selected. Some primers that were predicted to amplify the JHM strain but not the MHV-1 strain were included in the multiplex, to check for possible evolutionary change of the original sequence toward the annotated reference JHM sequence or cross reactions with primer-genome mismatches.

Samples from MHV-1 infected mice were provided by Dr. Richard Bowen at Colorado State University. The MHV-1 strain used to infect the mice was obtained from American Type Culture Collection (Manassas, VA) and viral stock was propagated in murine fibroblast 17Cl-1 cells then used to infect C3H mice via intranasal route. Mice were sacrificed four days after inoculation and bronchoalveolar lavage (BAL) fluid was collected. RNA was extracted from the BAL samples using Invitrogen TRIZOL reagent, as per the manufacturer's instructions. RNA was converted to cDNA using Superscript III (Invitrogen) and random hexamers according to the manufacturer's protocol.

Multiplexed primer sets were designed to cover the Nsp3 and 3′ genes with 3 primer pairs per genomic region amplified when possible (total number of primers tested in two multiplex reactions was 53, Table S1). The primers were tested in the lab first by testing the primer pairs in individual reactions then as multiplexed reactions. No effort was made to optimize the PCR cycling conditions. RT-PCR conditions were as follows: reverse transcription was performed using random hexamers and the Superscript III RT reverse transcriptase kit (Invitrogen). The MHV-1 cDNA templates were amplified using the Q5 Hot Start High-Fidelity DNA Polymerase kit (New England BioLabs, Ipswich, MA), following manufacturer's instructions. PCR conditions consisted of 98°C for 30 s, followed by 35 cycles of 98°C for 10 s, 60°C for 20 s, and 72°C for 1 min. The final cycle was 72°C for 2 min.

**Figure 2:**
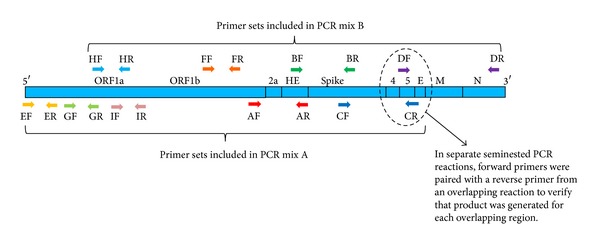
Diagram of the murine hepatitis virus (MHV) genome regions for which primer sets were tested. The approximate position of each region amplified by primer sets is shown (MHV genome is not drawn to scale). Each multiplex reaction consisted of primer sets that do not overlap in regions amplified. Each region is amplified using 3 forward primers and 3 reverse primers (Table S1; see Supplementary Material available online at http://dx.doi.org/10.1155/2014/101894). For example, the A primer set consists of 3 forward primers (A1F, A2F, and A3F) and 3 reverse primers (A1R, A2R, and A3R). To verify that each region is amplified in the multiplex reaction, a second set of seminested PCRs were performed using the amplicons from the multiplex reaction as a template. For example, to ensure region A was amplified, the PCR product from the A mix multiplex was diluted 1 : 10,000 and used as template in a PCR reaction with AR1 primer paired with BF2 (Table S2). Primers are labeled according to genome region (A-I) and primer direction (F = forward, R = reverse).

Two multiplex reactions were set up with each containing a group of nonoverlapping primer sets (Figure 2). For example, multiplex “A” included primer sets A, C, E, G, and I and multiplex “B” had primer sets B, D, F, and H. By staggering the primer sets into different multiplex reactions, the amplification of overlapping primer regions created by the reverse primer from one set with the forward primer of the overlapping, adjacent primer set was eliminated. Without this strategy, these overlapping primer sets would dominate the PCR reaction due to the small size of these amplicons.

The amplification of each primer pair in the multiplex was tested using a seminested PCR strategy to verify that the correct, specific amplicons were being produced from each multiplex of primers for a given region (Figure 2, Table S2). The multiplex PCR products served as templates for PCR reactions with primer pairs that included the reverse primer of one region paired with the forward primer from the downstream adjacent region to determine if the template generated from the multiplex was present. To ensure that the PCR product was generated from the multiplex product template rather than genomic DNA carried over from the initial sample, the multiplex product template was diluted 1 : 10,000 or excised from a gel and purified prior to use as a template.

## 3. Results and Discussion

All the primers for both (*s*, *x*) settings are provided as Supplementary data as are the predicted amplicon start and end positions in each target genome from a multiplex of the primers for a given viral target set. Tiled amplification of these viruses required from 2 to 116 primers (Table 1). Primers are predicted to be specific to the target organisms for the most part, although not exclusively (Tables 3 and 4). The few cases of off-target amplification come from closely related organisms in the same family such as Old World (OW) and New World (NW) Arenaviruses or other Flaviviruses amplified by the Japanese encephalitis virus (JEV) multiplex. The three exceptions were a single amplicon of 2830 bp from a BAC clone of Zea mays (maize) from the Ebola 3 kb multiplex, a single amplicon of 3610 bp from* Methylococcus capsulatus* str. Bath from the OW Arena S segment 3 kb multiplex and a single amplicon of 851 bp from a human BAC from a library at CalTech. All three of these predicted nontarget amplicons result from a single primer in each of those reactions performing as both forward primer (FP) and reverse primer (RP). Nonetheless, the primer multiplexes described here should strongly favor the preferential enrichment of desired targets.

Deriving each primer set required multiple sequence alignment and a call to run_tile_primers in the current PriMux software distribution (http://sourceforge.net/projects/primux/). In comparison, primer design with the JCVI pipeline for any of these target sets would require the following steps: (1) inspection of a phylogeny for the full target set to build multiple smaller clade-level sets with no more than 10% sequence variation, (2) realignment of the clade-level sets, (3) running of the JCVI pipeline on each clade set, (4) assessing which target sequences are not amplified after one design round and rerun the pipeline on those sequences for each clade, (5) and repeating step 4 until all target sequences are predicted to be amplified.

## 4. MHV Results 

Multiplexed primers were tested in the lab as primer pairs in individual reactions then as multiplexed reactions. Twenty-two of the primer pairs worked and four failed to give a product and were paired with other primers in subsequent testing or if necessary, replaced with an alternative primer. Amplicons were detected in the expected size ranges, confirming amplification of the expected regions from the multiplexed sets (Figure S1). In some cases extra bands were present, but they were generally smaller than the targeted size; this was common when the template cDNA was obtained from a clinical sample rather than high titer cell culture derived viral stock from this study. The PCR products generated with these highly multiplexed assays were then sequenced using Illumina ultradeep sequencing with a high fidelity polymerase. These primers yielded high coverage averaging 150,000x of the genomic regions amplified by the multiplex primers.

## 5. Conclusions

Software is described to generate tiled, multiplex, and degenerate amplification primers to span entire genomes or regions of many variant sequences. This tool should facilitate the amplification of overlapping products across whole genomes or user-specified regions of target sets with high levels of variation. Applications include target enrichment for viral discovery of new members in a viral family from a complex host background, improving high throughput sequencing sensitivity and coverage of a rapidly evolving virus, or enriched coverage of variants in a gene family.

## Supplementary Material

The data for the viral computational examples is given in two tarballs (TilingPrimers10kb.tar.gz, TilingPrimers3kb.tar.gz) containing subdirectories for each viral group, with text files containing the primers in fasta format and predicted hits in tab delimited text. The Supplementary information word document contains a detailed explanation of the output format of files provided in the tarballs, a listing of the MHV genomes and the regions file used in the MHV example, Table S1 containing the primers used for MHV-1 amplification, Table S2 with the primers, predicted product sizes, and PCR results used in the semi-nested PCR evaluation, and Figure S1 with the gel banding patterns from the multiplex amplification of MHV-1.

## Figures and Tables

**Table 1:** Summary of average lengths, number of sequences, and percentage of conserved bases in a multiple sequence alignment (with MUSCLE [5]), and number of tiled primers required for the short and long amplicon settings.

Organism	Number of sequences	Avg. Length	Consensus (%)	Number of primers for ~3,000 bp amplicons	Number of primers for ~10,000 bp amplicons
CCHF_S	56	1668	39	6	6
CCHF_M	49	5314	24	46	16
CCHF_L	31	12113	46	69	27
RVF_S	89	1684	53	2	2
RVF_M	69	3885	78	4	6
RVF_L	62	6404	83	6	4
Ebola	22	18659	5	116	35
Marburg	31	19115	70	34	8
Hendra	10	18234	97	12	4
Nipah	9	18247	91	18	6
Junin_L	12	7114	96	6	2
Machupo_L	5	7141	88	10	2
Junin_S	26	3410	80	4	4
Machupo_S	13	3432	76	4	4
JEV	144	10968	56	26	6
NW_Arena_S	100	3396	18	64	42
NW_Arena_L	42	7107	18	83	19
OW_Arena_S	54	3547	8	116	32
OW_Arena_L	45	7199	21	110	35
TBEV	67	10840	36	56	10

Abbreviations: CCHF = Crimean-Congo hemorrhagic fever, RVF = Rift Valley fever, JEV = Japanese encephalitis virus, NW_Arena = New World Arenavirus, OW_Arena = Old World Arenavirus, TBEV = tick-borne encephalitis virus, _L = L segment, _S = S segment.

**Table 2:** Parameters used for primer design in *in silico* examples and MHV example presented here.

	*In silico* primer settings	MHV primer settings
Primer length range	18–25	18–27
*T*_*m*_ range allowed^1^	60–65°C	58–65°C
Number degenerate bases allowed per primer	5	3
Minimum distance of degenerate base to 3′ end of primer	3 nt	3 nt
Minimum trimer entropy allowed (to avoid repetitive sequence)^2^	3.5	3.3
Maximum length of homopolymer allowed	4 nt	5 nt
GC% range allowed	20–80	20–80
Minimum primer dimer Δ*G*	−6 kcal/mol	−15 kcal/mol
Minimum hairpin Δ*G*	−5 kcal/mol	−12 kcal/mol
Primer selection iterations	1	3

^1^ *T*_*m*_ is calculated using Unafold [6].

^2^Low complexity regions (repetitive sequence) are excluded from consideration as primers by setting a minimum entropy threshold for a primer candidate. The entropy *S*_*i*_ of a sequence was computed by counting the numbers of occurrences of *n*_*AAA*_, *n*_*AAC*_,…, *n*_*TTT*_ of the 64 possible trimers in the probe sequence, and dividing by the total number of trimers, yielding the corresponding frequencies *f*_*AAA*_,…, *f*_*TTT*_. The entropy is then given by the sum of −*f*_*t*_log_2_⁡*f*_*t*_ where the sum is over the trimers *t* with *f*_*t*_ ≠ 0.

**Table 3:** Number of nontarget amplicons predicted in a multiplex reaction of tiled primers for 3 kb amplicons. In a multiplex of the 3 kb-amplicon tiled primers for a given organism, of the possible reactions producing products, only a small number of primer combinations are predicted to amplify regions in nontarget organisms. Counts show the number of unique primer combinations in a multiplex that yield products for any sequence in the NCBI nt nucleotide database. The numerator is for any nontarget organism in nt and the denominator is for any target or nontarget organism in nt, that is, nonspecific/total of the possible primer combinations in the multiplex predicted to yield product when compared against nt. Vastly more amplicons are produced from target organisms, indicating any contaminating nontarget species should be a small minority of amplified product.

Organism	Nontarget amplicons/total amplicons	Nontarget amplicon source organism
CCHF_S	0/160	—
CCHF_M	0/1934	—
CCHF_L	0/3753	—
RVF_S	0/137	—
RVF_M	0/356	—
RVF_L	0/753	—
Ebola	1/2657	Zea mays clone BAC ZMMBBb0342E21
Marburg	0/1511	—
Hendra	0/206	—
Nipah	0/286	—
Junin_L	0/69	—
Machupo_L	0/153	—
Junin_S	0/84	—
Machupo_S	0/32	—
JEV	7/9515	Rocio West Nile
NW_Arena_S	56/1543	Ippy Lassa Luna Lymphocytic choriomeningitis Mobala Mopeia
NW_Arena_L	0/819	—
OW_Arena_S	73/2509	Allpahuayo Amapari Bear canyon Chapare Cupixi Dandenong Flexal Guanarito Junin Latino Lujo Machupo *Methylococcus capsulatus* str. Bath Parana Pirital Sabia Tamiami Whitewater Arroyo
OW_Arena_L	1/1826	Dandenong
TBEV	0/4925	—

**Table 4:** Number of nontarget amplicons predicted in a multiplex reaction of tiled primers for 10 kb amplicons. As in Table 3, but for the multiplexes of the 10 kb-amplicon tiled primers.

Organism	Nontarget amplicons/total amplicons	Nontarget amplicon source organism
CCHF_S	0/160	—
CCHF_M	0/261	—
CCHF_L	0/253	—
RVF_S	0/137	—
RVF_M	0/487	—
RVF_L	0/195	—
Ebola	0/534	—
Marburg	0/123	—
Hendra	0/50	—
Nipah	0/74	—
Junin_L	0/12	—
Machupo_L	0/7	—
Junin_S	0/95	—
Machupo_S	0/32	—
JEV	0/1554	—
NW_Arena_S	1/337	Human chromosome 14 BAC C-2555K7 of library CalTech-D
NW_Arena_L	0/86	—
OW_Arena_S	0/316	—
OW_Arena_L	0/131	—
TBEV	0/189	—
