# Supplementary material for: Multiplex Degenerate Primer Design for Targeted Whole Genome Amplification of Many Viral Genomes
Source: Adv Bioinformatics. 2014 Aug 3;2014:101894. doi: 10.1155/2014/101894 (PMC4137498; doi:10.1155/2014/101894)
Supplement: Supplementary file 1 — The data for the viral computational examples is given in two tarballs (TilingPrimers10kb.tar.gz, TilingPrimers3kb.tar.gz) containing subdirectories for each viral group, with text files containing the primers in fasta format and predicted hits in tab delimited text. The Supplementary information word document contains a detailed explanation of the output format of files provided in the tarballs, a listing of the MHV genomes and the regions file used in the MHV example, Table S1 containing the primers used for MHV-1 amplification, Table S2 with the primers, predicted product sizes, and PCR results used in the semi-nested PCR evaluation, and Figure S1 with the gel banding patterns from the multiplex amplification of MHV-1. [file 101894.f1.docx]

**Supplementary information**

We computed 2 alternative primer sets for each viral target set, for short (3kb) and long (10 kb) amplicons.

The primers* files contain a fasta-formatted listing of the primers, where the headers have the following example format:

>0part_1|F

CGGGCYATCAATATGCTGAAAC

>0part_2|R

NGCYCTYTCAMGTTTCCA

>0part_3|F

ACTTCTTGGCTTAGTATCGTTGA

>0part_4|R

SSTCCAKCAMRCTCATCA

>0part_5|F

GAATCGAGAGATTAGTGCAGTTTA

>0part_6|R

TGCTTTTTGGTCCSGCTA

>1part_1|F

ATGGGHTGGAAAGCATGG

>1part_2|R

CTGGTTATGGGVGATGGGTTT

>1part_3|F

CYGAGACAAAGGAATGCCCT

>1part_4|R

ATGGTGGGYTTCACRCTC

The #part indicates the region of the alignment that primer pair targets (0part is the first region, 1part is the second region, etc.). The _# after the part label counts the primers in the multiplex needed to amplify sequence variants for that part. The list above shows that the first region of the alignment, 0part, requires 6 primers to amplify potential sequence variants in a sample, and 1part requires 4 primers. In multiplex, the primers can pair up in different combinations, acting as either forward or reverse primers depending on the partner. There may be an odd number of primers for a part if, for example, the same forward primer can amplify strains A and B, but different reverse primers are needed due to strain variation at the other end.

The product_positions and nt_hits files have the following tab-delimited columns:

FPid FPseq IOid IOseq RPid RPseq ID start stop length strand fullID

FPid and RPid are the sequence identifier (corresponding to the same identifier in the appropriate primer file) of the forward and reverse primers, and FPseq and RPseq are the forward and reverse primer sequence, respectively. The columns IOid and IOseq are empty since there are no TaqMan probes. ID represents an abbreviated strain name. Start and stop are the positions of the beginning and end of the amplicon in the indicated ID, and length is the length of the amplicon. Strand indicates which strand the FP-probe-RP are on (probe is NONE since it is not present). The fullID is the full sequence identifier. Predicted amplicons from the available target genomes are shown for amplicons of at least 500 bp, assuming all the primers in the set are combined in a single multiplex reaction and no mismatched hybridizations occur except for those specified by the degenerate bases in the primer sequences.

The files nt_hits contain similar information, only comparing primers to the nt database, and so contain nontargets amplified as well as targets. The product_positions files only contain target sequences. Both of these only show products in the length ranges of 500-7800 nt (primers for s=3 kb) or 500-26,000 nt (primers for s=10 kb).

The data for the viral computational examples is given in two tarballs (TilingPrimers10kb.tar.gz, TilingPrimers3kb.tar.gz) containing subdirectories for each viral group, with text files containing the primers in fasta format and predicted hits in tab delimited text.

MHV genomes

1 JHM_gi60115391 ref|NC_006852.1|gnl|NCBI_GENOMES|18238|gi|60115391|Murine hepatitis virus strain JHM, complete genome

2 ML-10_gi7769351 gi|7769351|gb|AF208067.1|AF208067 Murine hepatitis virus strain ML-10, complete genome

3 Penn_97-1_gi7769340 gi|7769340|gb|AF208066.1|AF208066 Murine hepatitis virus strain Penn 97-1, complete genome

4 A59_gi51557240 gb|AY700211.1|gi|51557240|Murine hepatitis virus strain A59, complete genome

5 MHV-MI_gi293330422 dbj|AB551247.1|gi|293330422|Murine hepatitis virus RNA, complete genome, strain: MHV-MI

6 S_gi298199702 gb|GU593319.1|gi|298199702|Murine hepatitis virus strain S, complete genome

7 MHV-JHM_IA_gi225403280 gnl|gcv|TCVSP-WEISS-00009|gb|FJ647226.1|gi|225403280|Murine coronavirus MHV-JHM.IA, complete genome

8 RA59_R13_gi225403195 gnl|gcv|TCVSP-WEISS-00001|gb|FJ647218.1|gi|225403195|Murine coronavirus RA59/R13, complete genome

9 RJHM_A_gi225403205 gnl|gcv|TCVSP-WEISS-00002|gb|FJ647219.1|gi|225403205|Murine coronavirus RJHM/A, complete genome

10 inf-MHV-A59_gi225403270 gnl|gcv|TCVSP-WEISS-00010|gb|FJ647225.1|gi|225403270|Murine coronavirus inf-MHV-A59, complete genome

11 repJHM_RA59_gi225403292 gnl|gcv|TCVSP-WEISS-00013|gb|FJ647227.1|gi|225403292|Murine coronavirus repJHM/RA59, complete genome

12 repA59_RJHM_gi225403227 gnl|gcv|TCVSP-WEISS-00005|gb|FJ647221.1|gi|225403227|Murine coronavirus repA59/RJHM, complete genome

13 SA59_RJHM_gi225403238 gnl|gcv|TCVSP-WEISS-00004|gb|FJ647222.1|gi|225403238|Murine coronavirus SA59/RJHM, complete genome

14 MHV-3_gi225403260 gnl|gcv|TCVSP-WEISS-00008|gb|FJ647224.1|gi|225403260|Murine coronavirus MHV-3, complete genome

15 MHV-1_gi225403250 gnl|gcv|TCVSP-WEISS-00007|gb|FJ647223.1|gi|225403250|Murine coronavirus MHV-1, complete genome

16 RA59_SJHM_gi225403217 gnl|gcv|TCVSP-WEISS-00003|gb|FJ647220.1|gi|225403217|Murine coronavirus RA59/SJHM, complete genome

17 A59_gi9629812 ref|NC_001846.1|gnl|NCBI_GENOMES|12745|gi|9629812|Murine hepatitis virus strain A59, complete genome

18 A59A59_B11_variant_gi226693969 gnl|gcv|TCVSP-WEISS-00011|gb|FJ884686.1|gi|226693969|Murine hepatitis virus strain A59 B11 variant, complete genome

19 A59A59_B12_variant_gi226693979 gnl|gcv|TCVSP-WEISS-00012|gb|FJ884687.1|gi|226693979|Murine hepatitis virus strain A59 B12 variant, complete genome

20 ML-11ML-11_RNA-directed_RNA_polymerase_orf1A_gi7739593 gi|7739593|gb|AF207902.1|AF207902 Murine hepatitis virus strain ML-11 RNA-directed RNA polymerase (orf1A), RNA-directed RNA polymerase (orf1B), non-structural protein (orf2A), hemagglutinin esterase protein (orf2B), spike glycoprotein precursor (orf3), non-structural protein (orf5A), envelope glycoprotein E (orf5B), matrix glycoprotein (orf6), and nucleocapsid protein (orf7) genes, complete cds

21 2_gi6625759 gi|6625759|gb|AF201929.1|AF201929 Murine hepatitis virus strain 2, complete genome

22 Betacoronavirus_MHV_S_3239-17_gi380706987 gb|JQ173883.1|gi|380706987|Murine hepatitis virus strain S/3239-17, complete genome

Regions file used for MHV example

Nsp1 0 1400

Nsp3 2400 9100

Nsp14 17500 20500

3primeGenes 21100 31000

Table S1: Primers used for MHV-1 amplification. Primers in bold are not predicted to amplify the MVH-1 strain we used due to the very short match lengths.

| **Primer** | **Primer Name** | **Primer length** | **Percent identity to MHV-1 over length of match** | **Length of match** | **Start** | **End** |
| --- | --- | --- | --- | --- | --- | --- |
| AATCCATTTGTGAGYGACAG | A1F | 20 | 95 | 20 | 21091 | 21110 |
| ATACAAACAGGYGCCATA | A1R | 18 | 94 | 18 | 23747 | 23730 |
| ATTATGGAARTTGTATAACCTTACCC | A2F | 26 | 96 | 26 | 21122 | 21147 |
| **AAATGCACCCTGCTGGGC** | **A2R** | **18** | **100** | **12** | **23655** | **23644** |
| GATAAGGRTGTTGCMCCTG | A3F | 19 | 89 | 19 | 21013 | 21031 |
| TACCCATAGGCTGGCCAA | A3R | 18 | 100 | 18 | 23696 | 23679 |
| ATGGTGATTTTCAYTTCAGGC | B1F | 21 | 95 | 21 | 23579 | 23599 |
| **GAAGCGCCTCATCCGTGC** | **B1R** | **18** | **100** | **8** | **27537** | **27544** |
| CNTGTCAGCTGCCATATT | B2F | 18 | 100 | 16 | 23509 | 23524 |
| ACTGTAAATGGTTCAAAAGTAGTTA | B2R | 25 | 100 | 25 | 26193 | 26169 |
| GTCTGACAATATGACCGCTG | B3F | 20 | 100 | 20 | 23484 | 23503 |
| ACTACCCATACGBARATCAC | B3R | 20 | 90 | 20 | 26104 | 26085 |
| **AATATTTCCCGTGAGGARAACC** | **C1F** | **22** | **100** | **11** | **25994** | **26004** |
| GGTTGCTACACCAMGCCT | C1R | 18 | 94 | 18 | 28606 | 28589 |
| TTATTTGGGYTGTGTTGTTAATGC | C2F | 24 | 96 | 24 | 26029 | 26052 |
| TTGCTACACCAAGCCTTTAAA | C2R | 21 | 100 | 21 | 28604 | 28584 |
| **CGAACCGGCTCTRMTCTA** | **C3F** | **18** | **100** | **7** | **3647** | **3641** |
| AGGCCGTGAAGCTAGTAA | C3R | 18 | 94 | 18 | 28687 | 28670 |
| CWTGTGAGTGAYGCHTGG | D1F | 18 | 88 | 16 | 28473 | 28488 |
| TTAGATCCAAAGAAGAAGGCAC | D1R | 22 | 100 | 22 | 30724 | 30703 |
| **TGASACCACCAGCTACAT** | **D2F** | **18** | **92** | **13** | **28443** | **28455** |
| GAATGGGGAACTGYGGAT | D2R | 18 | 94 | 18 | 30678 | 30661 |
| ACGCMGVACGCGDGACTT | D3F | 18 | 83 | 18 | 28491 | 28508 |
| TGGGTYCATCAGCACCAC | D3R | 18 | 89 | 18 | 30768 | 30751 |
| AACTGYGGATCAYTAGTTCCAAGTTT | D4R | 26 | 92 | 26 | 30749 | 30774 |
| AACCTAATGTCTTGCCAAAAACT | G2R | 23 | 100 | 23 | 5105 | 5083 |
| TRTGTCTTGCTGGCAGTA | G1F | 18 | 100 | 16 | 2378 | 2393 |
| AACYTTGGTKACATTTATRCCATC | G3R | 24 | 88 | 24 | 5140 | 5117 |
| AAGATGATGTTGTTGATGTGGTTA | G3F | 24 | 100 | 24 | 2496 | 2519 |
| CCCTTGTGAATGGCACTA | G1R | 18 | 100 | 18 | 5163 | 5146 |
| TGTTGATGTGGTYAAAGCC | G2F | 19 | 94 | 17 | 2506 | 2522 |
| TAAGGGCTTTACTYGCTA | H1F | 18 | 94 | 18 | 4998 | 5015 |
| RAATCGCAGTAACGTAAAAGCTG | H1R | 23 | 100 | 22 | 7527 | 7506 |
| GWGGCGAGTGTYAGCCAA | H2F | 18 | 94 | 16 | 4981 | 4996 |
| TAGCTGTCACCACGATGT | H2R | 18 | 100 | 18 | 7547 | 7530 |
| TTGATAGTGTTGATGGTGTTAGAA | H3F | 24 | 100 | 24 | 4869 | 4892 |
| MACAAACAAACGAGCGCT | H3R | 18 | 100 | 17 | 7485 | 7469 |
| MATGCAGTTRYTGACCAC | I2F | 18 | 88 | 17 | 7412 | 7428 |
| CRAGACCAAAHGTAGACTCATAC | I2R | 23 | 95 | 21 | 8898 | 8878 |
| TTTATGCTGGRGACYATGC | I1F | 19 | 89 | 19 | 7445 | 7463 |
| DACMGCAACCACAACAGG | I1R | 18 | 94 | 17 | 8943 | 8927 |

Table S2: Primers used for a semi-nested PCR to evaluate amplification for primer sets used for MHV-1 amplification (as illustrated in Figure 2).


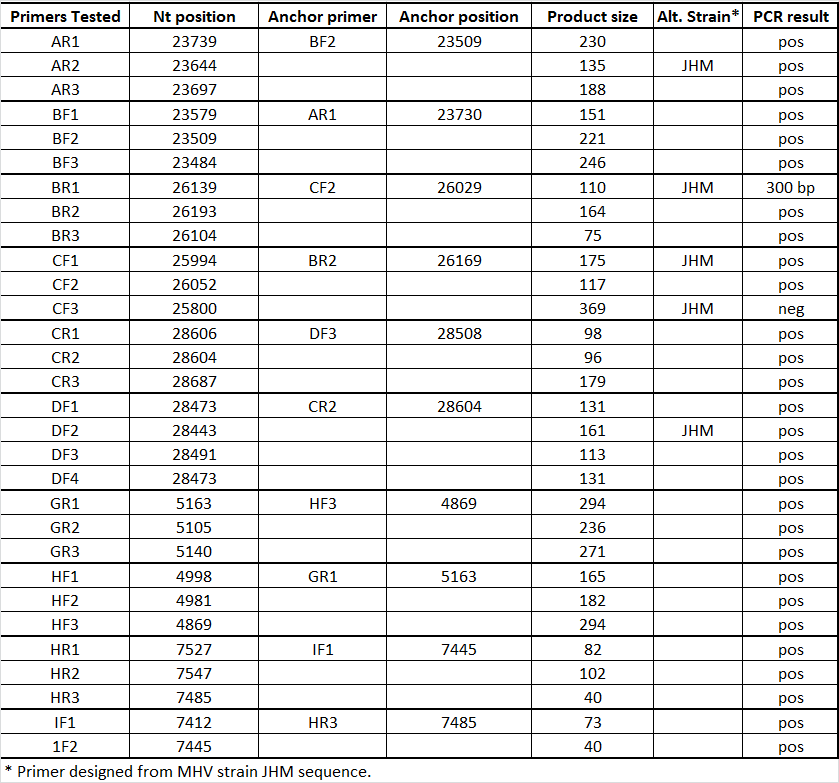

**Suppl. Figure 1. Highly multiplexed amplification of MHV-1.**

MHV-1 cDNA was generated from viral RNA extracted individually from BAL of four MHV-1 infected mice. Samples were PCR amplified using three different primer mixes (A, B and I). The PCR products (5 µl) were resolved on a 0.8% agarose gel using 1Kb Plus DNA E-gel ladder (M). Primer mixes A and B yielded the expected 2.5Kb PCR product and primer mix I yielded the expected 1.5Kb product. Although semi-nested PCR results showed that all primer sets yielded products as part of the A multiplex, I primers were run separately due to concerns that the smaller product size of the I primer sets would decrease yield of the other primer sets. The corresponding PCR products are as follows: primer mix A lanes 3, 6, 9, 12 and 15; primer mix B lanes 1, 4, 7, 10, 13 and16; primer mix I lanes 2, 5, 8, 11, and 14.
